# Protective effects of *Ficus carica* leaves on glucose and lipids levels, carbohydrate metabolism enzymes and β-cells in type 2 diabetic rats

**DOI:** 10.1080/13880209.2017.1279671

**Published:** 2017-02-13

**Authors:** Santiagu Stephen Irudayaraj, Sunil Christudas, Stalin Antony, Veeramuthu Duraipandiyan, Al-Dhabi Naif Abdullah, Savarimuthu Ignacimuthu

**Affiliations:** a Division of Ethnopharmacology, Entomology Research Institute, Loyola College, Chennai, India;; b Division of Bioinformatics, Entomology Research Institute, Loyola College, Chennai, India;; c Department of Botany and Microbiology, Addiriyah Chair for Environmental Studies, College of Science, King Saud University, Riyadh, Saudi Arabia;; d Visiting Professor Programme, Deanship of Scientific Research, College of Science, King Saud University, Riyadh, Saudi Arabia

**Keywords:** Ficusin, hypolipidemic, hypoglycemic

## Abstract

**Context:** The decoctions of *Ficus carica* Linn. (Moraceae) leaves are used in the folklore treatment of diabetes.

**Objective:** To evaluate the effect of *F. carica* on glucose and lipids levels, carbohydrate metabolism enzymes and β-cells protective effects in type 2 diabetes.

**Material and methods:** Diabetes was induced in 15 days high-fat diet (HFD)-fed Wistar rats by intraperitoneal injection of streptozotocin (STZ) (40 mg/kg). The ethyl acetate extract (250 and 500 mg/kg) of *F. carica* leaves was administered for 28 days. Oral glucose tolerance (OGTT) and intraperitoneal insulin tolerance tests (ITT) were evaluated on 15th and 25th days, respectively.

**Results:** The ethyl acetate extract (250 and 500 mg/kg) of n *F. carica* leaves showed significant effect (*p* < 0.005) in the levels of blood glucose, total cholesterol (TC), triglycerides (TG), body weight and hepatic glycogen. In OGTT, *F. carica* (250 and 500 mg/kg) significantly (*p* < 0.005) detained the increase in blood glucose levels at 60 and 120 min and in ITT, *F. carica* enhanced the glucose utilization significantly (*p <* 0.005) over 30 and 60 min compared to diabetic control. Further, the altered activities of key carbohydrate metabolizing enzymes such as glucose-6-phosphatase, fructose-1,6-bisphosphatase and hexokinase in the liver tissue of diabetic rats were significantly (*p* < 0.005) reverted to near normal levels upon treatment with *F. carica*. Immumohistochemical studies of islets substantiated the cytoprotective effect on pancreatic β-cells.

**Discussion and conclusions:***F. carica* leaves exerted significant effect on carbohydrate metabolism enzymes with promising hypoglycemic and hypolipidemic activities in type 2 diabetic rats.

## Introduction

Type 2 diabetes mellitus (T2DM) or non-insulin-dependent diabetes mellitus accounts for more than 90–95% of world human diabetic population (Naik et al. 2013). Insulin resistance, defective insulin secretion and pancreatic β-cell damage were observed as metabolic features of T2DM. It is predicted that by 2030 the total number of people having diabetes will reach 366 million (Shaw et al. 2010). Along with high blood glucose level (hyperglycemia) and variation in the levels of serum lipids, diabetes will lead to micro- and macro-vascular complications. These complications are the major cause of morbidity and death in diabetic subjects (Sunil et al. 2012a). Insulin, oral hypoglycemic drugs like sulphonylurea derivatives, biguanides, thiazolidinediones and α-glucosidase inhibitors are currently used to treat type 2 diabetes. These agents have undesirable side effects: thiazolidinediones induce obesity, osteoporosis and sodium retention; sulphonylurea derivatives produce hypoglycemia and the biguanide (metformin) puts patients at the risk of developing lactic acidosis (Surya et al. 2014). Medicinal plants are used traditionally in many countries to control diabetes. Consequently, many medicinal plants have been investigated with the aim of discovering potential hypoglycemic agents (Sunil et al. 2012b). Because of its activity, nontoxic, with little or no side effects, World Health Organization (WHO) has recommended that traditional plants can be an excellent candidate for the treatment of diabetes (Stephen Irudayaraj et al. 2012). Plants used in traditional medicine to treat diabetes mellitus represent a valuable alternative for the control of this disease.

*Ficus carica* Linn. (Moraceae), commonly referred as Figs or Anjir, is cultivated in tropical and subtropical regions of India and worldwide for their nutritive and medicinal properties; it has been widely used in indigenous systems of medicine, such as ayurveda, siddha and homoeopathy (Manda et al. 1997). *F. carica* leaves, bark, tender shoots, fruits, seeds and latex are traditionally used in the treatment of jaundice, diarrhea, nutritional anemia and as an anti-inflammatory agent (Canal et al. 2000). Decoctions of *F. carica* leaves are used as folk medicine for the treatment of diabetes (Perez et al. 1996). Hepatoprotective, hypoglycemic, antifungal, antispasmodic, antipyretic, anthelmintic, antioxidant and antimutagenic activities have been reported (Vikas & Vijay 2011). The antidiabetic property of *F. carica* has been reported in alloxan-induced insulin-deficient diabetic rat model (Stalin et al. 2012). The antihyperlipidemic effect of *F. carica* in high-fat diet and Triton X-100-induced hyperlipidemic animal models are reported (Dayanand & Kishanchandra 2014; Joerin et al. 2014). Therefore, this study was undertaken to find the effect of *F. carica* on glucose and lipids levels, carbohydrate metabolism enzymes and β-cells protective effects in HFD-fed STZ-induced type 2 diabetic rats.

## Materials and methods

### Chemicals and reagents

Streptozotocin (STZ) and all fine chemicals were purchased from Sigma-Aldrich Chemical Company (St. Louis, MO). Ultrasensitive rat insulin ELISA kit was obtained from Crystal Chem, Inc. (Downers Grove, IL). Glibenclamide and all other chemicals of analytical grade were purchased from local firm (Chennai, Tamil Nadu, India).

### Plant material

*F. carica* leaves were collected from Western Ghats, Tamil Nadu, India, during January 2015 and were duly authenticated by Ayyanar, the taxonomist of Entomology Research Institute, Loyola College (Voucher no. ERI/ETHPH/FC/234).

### Extraction of crude extracts of *F. carica*

*F. carica* leaves were rinsed with water, shade-dried and powdered. Fine powder (3 kg) was soaked in 9 L of hexane, ethyl acetate and methanol, sequentially for 72 h at room temperature. The extrats were filtered and concentrated using rotary evaporator at 40–55 °C. 42, 36 and 19 g were yielded from the hexane, ethyl acetate and methanol extracts, respectively, and they were stored in refrigerator at 4 °C. In our preliminary studies among hexane, ethyl acetate and methonal extracts, the ethyl acetate extract of *F. carica* showed highly significant glucose lowering activity (data not shown). Hence, the study was designed to investigate the hypoglycemic, hypolipidaemic and β-cells protective effects of the ethyl acetate extract of *F. carica* leaves in HFD-fed STZ-induced type 2 diabetic rats.

### HPLC-DAD analysis

Qualitative and quantitative analyses of major constituent/constituents in *F. carica* were carried out on Waters Alliance 2695 separations Module HPLC system with photodiode array detector (Waters, Milford, MA, 2996), with the reversed phase LC column, YMC pack ODS A (150 mm ×4.6 mm, 3.5 μm) was eluted with 0.1% orthophosphoric acid (A) and acetonitrile (B) in the ratio of 1:1 (A:B, v/v) as mobile phase system with a flow rate of 1 mL/min. The samples were injected using a 20 μL loop (Rheodyne, Rohnet Park, CA) and the separations were monitored with PDA signal at 270 nm. Peak purity was checked and the quantification was done. Ficusin (HPLC purity 98.6%) was used as standard and run at five concentrations (3–150 μg/mL) and should be linear in the range with a correlation coefficient (*r*^2^) of 0.99700. Using linear regression analysis, the ficusin content was estimated using the calibration cure appearing in the standard chromatogram using the following equation:

$$C\left(c\right)=\frac{A\left(c\right)}{A\left(\mathrm{st}\right)}\times C\left(\mathrm{st}\right)$$

where C(c) is the concentration of the constituent in the sample, A(c) is the peak area of the constituent in the sample chromatogram, C(st) is the concentration of the standard in the reference solution and A (st) is the area of the peak for the standard in the reference chromatogram (Vinholes et al. 2011).

### Experimental animals

Healthy male albino Wistar rats (170–190 g) were housed and raised at the animal housed of Entomology Research Institute in favourable environmental conditions at temperature (22 ± 2 °C), a relative humidity (45 ± 5 °C) and 12/12 h day/night cycle of 7 days. The animals were fed *ad libitum* with normal laboratory standard pelleted diet purchased from Sai Durga Feeds and Foods, Bangalore. The animal experiments and protocols were approved by the Institutional Animal Ethics Committee (IAEC-ERI-LC-05/13).

### Fixation of doses and acute toxicity study

Overnight fasted rats were divided into five groups of six rats each. *F. carica* was dissolved in vehicle (0.9% sodium chloride, 0.5% carboxymethyl cellulose (CMC) and 0.2% tween 80 in distilled water) and used for treatment. Treated rats were given oral doses of *F. carica* (1000, 2000, 3000, 4000, 5000 mg/kg) respectively. The control group was given vehicle alone. The physical signs of toxicity, such as writhing, gasping, palpitation and decreased respiratory rate, body weight changes, food and water consumption or mortality were observered for 1 h continuously, then for 4 h and finaly after every 24 h up to 14 days.

Neither mortality nor adverse effects except for mild writhing were observed up to 5000 mg/kg of *F. carica* extract. Hence, 250 and 500 mg/kg doses were preferred for consequent experiments (Oliveira et al. 2008).

### Development of T2DM

All the experimental rats were fed with HFD except normal control composed of standard rat pelleted diet 68%, dalda (saturated fat) 30%, and cholesterol 2% for 15 days. Single intraperitoneal injection of freshly prepared solution of STZ (40 mg/kg) in 0.1 M citrate buffer (pH 4.5) was used to induce diabetes. Rats were given 20% glucose solution for 24 h to prevent initial drug-induced hypoglycemic mortality. After seven days of STZ administration, blood samples were collected from the tail vein and plasma glucose levels were determined as described by Trinder (1969). Hyperglycemia was considered by measuring the fasting blood glucose (FBG) level. Rats with a FBG level above 250 mg/dL were categorized as diabetic and included in the study.

Experimental design

The animals were assigned into five groups of six animals in each group.

Group I: Normal control rats treated with vehicle alone.

Group II: Diabetic control rats treated with vehicle alone.

Group III: Diabetic rats treated with *F. carica* (250 mg/kg).

Group IV: Diabetic rats treated with *F. carica* (500 mg/kg).

Group V: Diabetic rats treated with glibenclamide (0.6 mg/kg).

Treatments (either vehicle or test drugs) were administered orally, once daily using intragastric tube for 28 days.

### Biochemical analysis

FBG and body weights of days 0, 14, 21 and 28 were measured. Plasma insulin (Ultrasensitive rat insulin ELISA kit), TC (Henley 1957) and TG (Foster & Dunn 1973) were determined on days 0 and 28. OGTT and ITT were performed on days 15 and 25, respectively. On 28th day, the animals were anesthetized and sacrificed by cervical decapitation. Blood was collected in dry EDTA containing test tubes and the liver tissues were sliced quickly and washed in ice cold saline and stored at −70 °C in liquid nitrogen for further analysis of glycogen. The homogenate prepared in ice-chilled 10% potassium chloride in 0.1 M phosphate buffer (pH 6.5) solution was used to measure the activities of hexokinase (Brandstrup et al. 1957), glucose-6-phosphatase (Koide & Oda 1959), fructose-1,6-bisphosphatase (Gancedo & Gancedo 1971) and hepatic glycogen (Van Handel 1965), respectively.

### Immunohistochemical analysis

Immunohistochemical study was done as described by Gandhi et al. (2012).

### Statistical analysis

The results were presented as mean ± SEM. Statistical analyses of all the data obtained were evaluated using one-way ANOVA followed by Student’s *t*-test (SPSS Program; Version 11.5, Chicago, IL). The differences were considered as significant at *p* < 0.005.

## Results

### HPLC-DAD analysis

**Figure 1.**
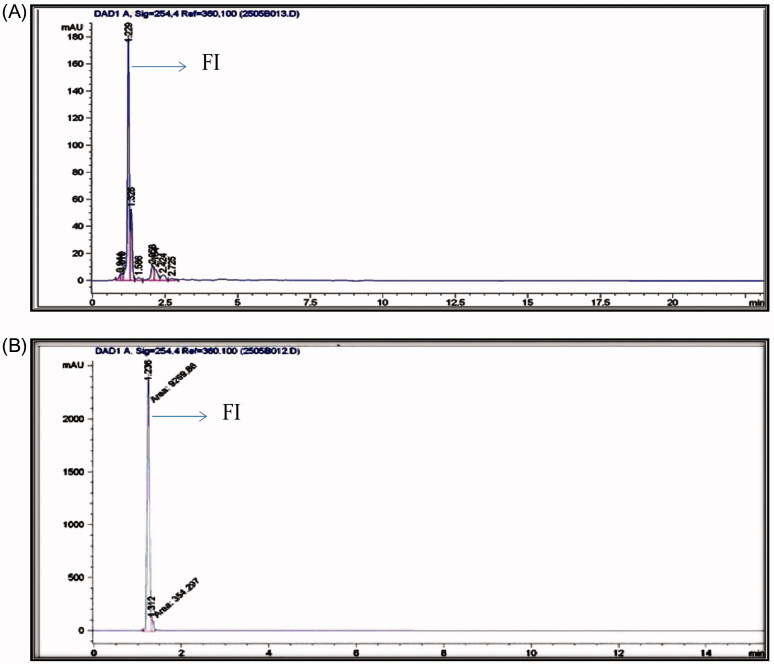
HPLC fingerprint chromatogram of *F. carica* with ficusin (A) and the standard chromatogram of ficusin (B) peak. FI: ficusin.

Quantification of ficusin (a furocoumarin) by HPLC analysis showed its content to be 6.46% w/w. The retention time (tR) of reference standard ficusin was observed at 1.2 min and it was found to be the same with ficusin present in the ethylacetate extract of the leaves of *F. carica* (Figure 1).

### Effect of *F. carica* on OGTT and ITT

**Figure 2.**
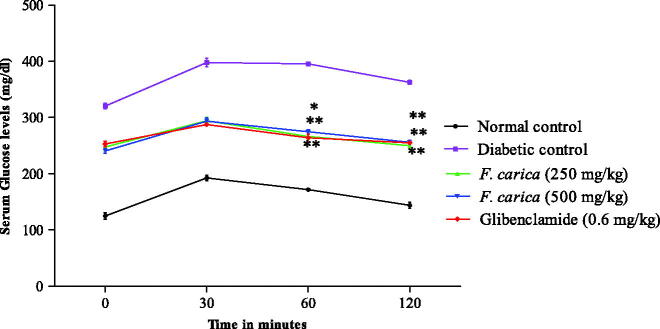
Effect of *F. carica* on 15th day OGTT.

**Figure 3.**
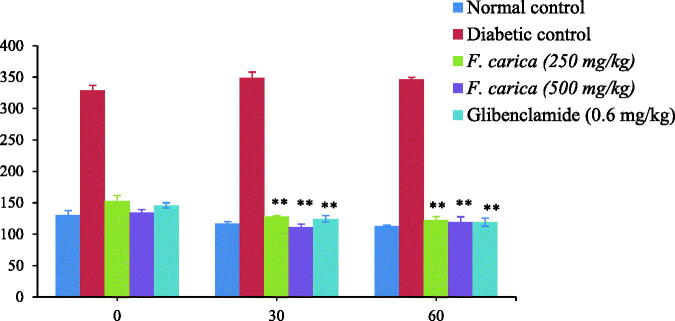
Effect of *F. carica* on 25th day ITT.

In oral glucose tolerance test, soon after glucose administration, the plasma glucose levels reached the maximum at 30 min in all the groups. Treatment with *F. carica* (250 and 500 mg/kg), significantly (*p* < 0.005) detained the increase of blood glucose levels at 60 and 120 min (Figure 2). In addition, *F. carica* treatment (250 and 500 mg/kg) enhanced the glucose utilization significantly (*p <* 0.005) over the complete period during the ITT compared to diabetic control rats (Figure 3).

### Effect of *F. carica* on FBG and body weight

There was a significant (*p <* 0.005) increase in the levels of FBG in HFD-fed STZ-treated diabetic control rats. *F. carica* (250 and 500 mg/kg) treatment significantly (*p <* 0.005) reduced the blood glucose level to near normal compared to glibenclamide-treated (0.6 mg/kg) rats (Table 1). The body weight was also increased in *F. carica-*treated (250 and 500 mg/kg) diabetic rats (Table 2).

**Table 1.** Levels of glucose in normal and diabetic rats after 28 days of treatment with *F. carica*.

	Fasting blood glucose levels (mg/dl)			
Groups	0 day	14th day	21st day	28th day
Normal control	77.82 ± 1.93	82.03 ± 2.11	79.21 ± 4.24	80.55 ± 2.18
Diabetic control	283.26 ± 7.82	319.4 ± 8.42	323.28 ± 3.64	336.35 ± 6.14
Diabetic + *F. carica* (250 mg/kg)	298.31 ± 6.69	249.67 ± 8.12**	174.81 ± 7.42**	137.27 ± 7.24**
Diabetic + *F. carica* (500 mg/kg)	274.37 ± 4.18	251.6 ± 7.44**	142.65 ± 8.12**	129.14 ± 8.23**
Diabetic + Glibenclamide (0.6 mg/kg)	283.61 ± 9.14	256.1 ± 6.72**	152.66 ± 7.74**	131.32 ± 9.13**

Values indicate mean ± standard error of the mean (SEM) of six rats per group.

** *p* < 0.005, compared with diabetic control values.

**Table 2.** Effect of *F. carica* on body weight after 28 days of treatment.

	Body weight (g)			
Groups	0 day	14th day	21st day	28th day
Normal control	188.12 ± 11.42	185. 2 ± 12.54	190.20 ± 12.10	190.22 ± 3.21
Diabetic control	209.25 ± 18.24	242.15 ± 10.71	264.16 ± 10.17	281.22 ± 13.52
Diabetic + *F. carica* (250 mg/kg)	180.48 ± 9.69	192.67 ± 9.12**	194.65 ± 8.56**	195.26 ± 9.12**
Diabetic + *F. carica* (500 mg/kg)	190.34 ± 10.18	195.4 ± 12.92**	201.21 ± 9.57**	204.46 ± 8.71**
Diabetic + Glibenclamide (0.6 mg/kg)	202.72 ± 9.14	209.8 ± 17.42**	215.89 ± 9.12*	219.51 ± 8.22**

Values indicate mean ± standard error of the mean (SEM) of six rats per group.

** *p* < 0.005, compared with diabetic control values.

### Effect of *F. Carica* on plasma insulin, TC and TG

Plasma insulin level showed a significant increase (*p <* 0.005) in untreated diabetic rats. After treatment with *F. carica* (250 and 500 mg/kg), plasma insulin level significantly (*p <* 0.005) reduced to normal when compared with the diabetic control rats. Similarly, increased TC and TG levels were observed in untreated diabetic rats. After treatment with *F. carica* (250 and 500 mg/kg) for 28 days, TC and TG levels were significantly (*p <* 0.005) reverted to the near normal level (Table 3).

**Table 3.** Effect of *F. carica* on Plasma insulin, TC and TG after 28 days of treatment.

	Plasma Insulin (μU/mL)		TC (mg/dl)		TG (mg/dl)	
Groups	0 day	28th day	0 day	28th day	0 day	28th day
Normal control	16.86 ± 1.74	16.28 ± 2.0	78.56 ± 5.81	79.14 ± 3.54	74.78 ± 3.42	71.65 ± 4.42
Diabetic control	22.45 ± 1.54	25.31 ± 0.46	99.24 ± 6.54	126.62 ± 7.88	149.74 ± 7.14	169.14 ± 6.12
Diabetic + *F. carica* (250 mg/kg)	24.52 ± 1.78	20.77 ± 0.84**	96.67 ± 5.32	88.14 ± 1.89**	163.78 ± 9.18	106.84 ± 5.23**
Diabetic + *F. carica* (500 mg/kg)	22.84 ± 1.64	15.84 ± 0.76**	96.98 ± 8.12	71.52 ± 4.64**	164.83 ± 6.23	94.29 ± 11.68**
Diabetic + Glibenclamide (0.6 mg/kg)	23.56 ± 1.51	16.13 ± 0.53**	91.26 ± 1.76	68.18 ± 4.58**	157.18 ± 7.11	89.18 ± 8.16**

Values indicate mean ± standard error of the mean (SEM) of six rats per group.

** *p* < 0.005, compared with diabetic control values.

### Effect of *F. Carica* on hexokinase, glucose-6-phosphatase, fructose-1,6-bisphosphatase and hepatic glycogen

The activities of hexokinase, glucose-6-phosphatase and fructose-1,6-bisphosphatase and the level of glycogen content in the liver of normal and diabetic rats are shown in Table 4. A significant (*p <* 0.005) increase in the activities of glucose-6-phosphatase and fructose- 1,6-bisphosphatase with significant (*p <* 0.005) decrease in hexokinase and glycogen were observed in diabetic rats when compared to normal control rats. Oral administration of *F. carica* (250 and 500 mg/kg) for 28 days significantly decreased the glucose-6-phosphatase and fructose-1,6-bisphosphatase, and significantly increased the hexokinase and glycogen to near normal.

**Table 4.** Effect of *F. carica* on hexokinase, glucose-6-phosphatase, fructose-1, 6-bisphosphatase and hepatic glycogen after 28 days of treatment.

Groups	Hexokinase (U/mg protein /min)	Glucose-6- phosphatase (U/mg protein/min)	Fructose-1,6- bisphosphatase (U/mg protein/min)	Hepatic glycogen (mg/g tissue)
Normal control	8.13 ± 1.74	0.29 ± 0.12	0.39 ± 0.07	24.74 ± 2.84
Diabetic control	4.98 ± 0.98	0.61 ± 0.12	0.69 ± 0.15	9.54 ± 1.82
Diabetic + *F. carica* (250 mg/kg)	6.28 ± 1.67*	0.37 ± 0.08**	0.53 ± 0.08*	18.58 ± 2.92**
Diabetic + *F. carica* (500 mg/kg)	7.74 ± 1.93**	0.26 ± 0.16**	0.42 ± 0.06**	20.69 ± 1.74**
Diabetic + Glibenclamide (0.6 mg/kg)	7.18 ± 1.24**	0.22 ± 0.05**	0.41 ± 0.07**	18.27 ± 1.22**

Values indicate mean ± standard error of the mean (SEM) of six rats per group.

* *p* < 0.05,

** *p* < 0.005, compared with diabetic control values.

### Immunohistochemical observations

**Figure 4.**
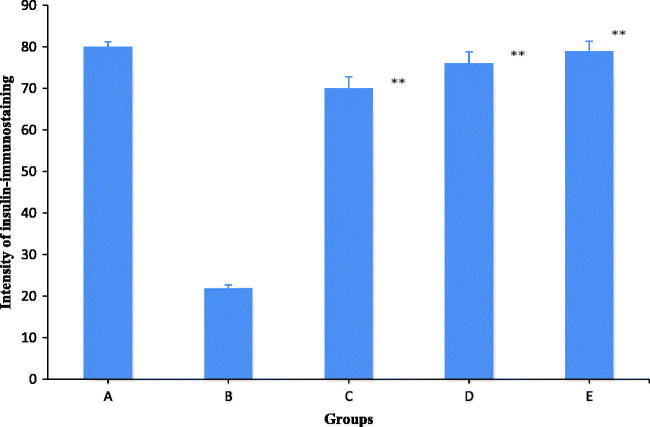
Histogram showing intensity of insulin-immunostaining expression of β-cells in islet of Langerhans. (A) Normal control. (B) Diabetic control. (C and D) Diabetic + *F. carica* (250 and 500 mg/kg b wt.). (E) Diabetic + glibenclamide (0.6 mg/kg). Values represent the mean ± standard error of the mean (SEM) of six rats per group. **p < 0.005, compared with diabetic control values.

**Figure 5.**
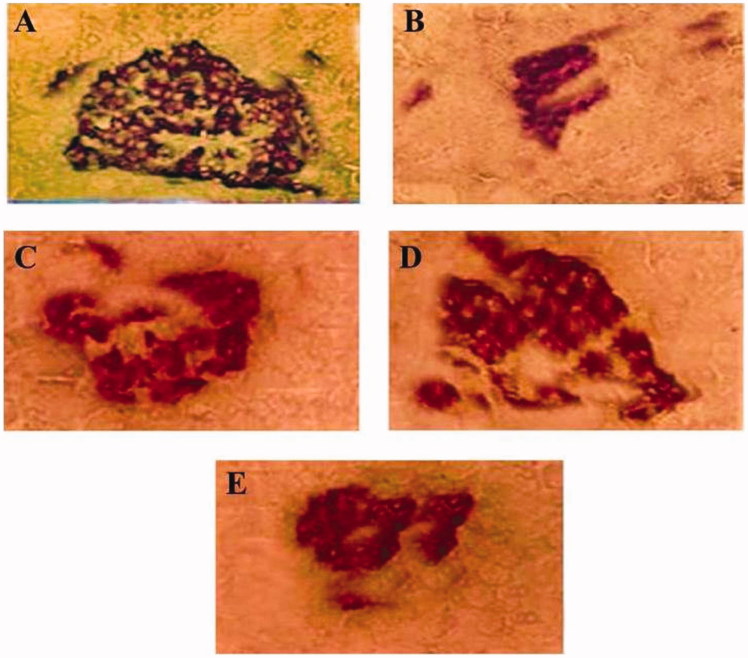
Light photo micrographs of rat pancreas showing insulin-immunoreative expression of β-cells in islet of Langerhans. (A) Islet of normal control rat. (B) Islet of diabetic control rat. (C and D) Islet of *F. carica-*treated (250 and 500 mg/kg) diabetic rats. (E) Islet of glibenclamide-treated (0.6 mg/kg) diabetic rats.

The results of the intensity of insulin-immunostaining expression is shown in Figure 4. Diabetic control rats showed a significant (*p* < 0.005) reduction in the level of insulin-immunostaining expression as compared to normal control rats. *F. carica-*treated (250 and 500 mg/kg) diabetic groups exhibited significant (*p* < 0.005) increase in the insulin-immunostaining expression compared to the vehicle-received diabetic control group. The immumohistochemical examinations showed islets with positive insulin-immunoreactive expression in the pancreas of normal rats (Figure 5(A)). Diabetic rats showed low amount of insulin-immunoreactive expression (Figure 5(B)). There was a relative rise in the expression of insulin-immunoreactivity with reliable dark brown insulin-immunostaining in the *F. carica-* (250 and 500 mg/kg) and glibenclamide-treated (0.6 mg/kg) diabetic groups compared with the diabetic control group (Figure 5(C–E)).

## Discussion

T2DM is a progressive and complex disorder that is emerging as a crucial threat to human population (Steppan et al. 2001). We observed in our study that HFD treatment along with intraperitoneal administration of STZ (40 mg/kg) damaged the insulin-secreting β-cells of the pancreas by breaking the DNA strand and caused depressed β-cell function, decreased endogenous insulin release and defects in insulin action (Koning et al. 2008). Other investigators have also used similar experimental T2DM animal model to explore drug discoveries (Zhang et al. 2010; Veerapur et al. 2012).

OGTT and ITT were performed on 15th and 25th day, respectively. The *in vivo* peripheral insulin action and insulin resistance in animals can be assed by OGTT, hence it is a simple and widely accepted method (Liou et al. 2002). In our study, *F. carica* stimulated the glucose uptake into peripheral tissues of the diabetic rats in a dose-dependent manner. ITT is useful to assess insulin sensitivity by exogenous administration of insulin. *F. carica* (250 and 500 mg/kg) reduced the blood glucose significantly at 30 and 60 min, suggesting imporved insulin sensitivity possibly by improving one or more defects, namely insulin receptor, insulin receptor substrate, glucose transporters or enzymes involved in phosphorylation of glucose (Benwahhoud et al. 2001; El Hilaly & Lyoussi 2002). These results from ITT and OGTT tests clearly established that *F. carica* administration reduced the glucose level by the mechanism of insulin-sensitized glucose uptake. Menaka et al. (2010) has also been reported in his study that, the similar mechanism of insulin-sensitized glucose uptake has been observed in ITT and OGTT of *Sida rhomboidea* extract on the C57BL/6J mice model.

A significant decrease in blood glucose and normalization of plasma insulin levels were observed in diabetic rats treated with *F. carica*. This could be due to the potentiation of the extract on pancreatic secretion of insulin from regenerated β-cells, or its action to release bound insulin from regenerated β-cells by inhibiting ATP-sensitive K^+ ^channels like glibenclamide (Sunil et al. 2012b). Yuan et al. (2001) have suggested that hyperinsulinaemia caused by insulin resistance in peripheral tissues damages the structural integrity of β-cells. In our study, the immunohistochemical analysis indicates that diabetic rats showed impaired and degranulated β-cells with the decrease of insulin-positive staining β-cells. But, *F. carica* treatment preserved the β-cell mass on diabetic rats. The extensive increase in insulin-immunoreactive expression in *F. carica-*treated diabetic rats substantiated the protective role of *F. carica* in the reversion of pancreatic β-cell damage caused by HFD-STZ. Coumarins are reported as potential hypoglycemic agents with insulin sensitization effect (Liang et al. 2009; Qin et al. 2010). *F. carica* contains good amount of furocoumarin, ficusin which could be responsible for its insulin sensitization effect. Moreover, ficusin has been reported as good antioxidant, antilipidemic and antidiabetic effects with their effects on GLUT4 translocation and PPARγ expression in type 2 diabetic rats (Stephen Irudayaraj et al. 2016).

The administration of HFD has significantly increased the TC and TG in the diabetic rats. In the present study, HFD-fed STZ-induced diabetic rats have also shown marked rise in their body weights compared to normal control rats. Diabetic condition with profound alterations in lipid profile leads to the development of atherosclerosis (Warnholtz et al. 2001). After the oral administration of *F. carica* for 28 days to diabetic rats, there was a significant restoration in altered lipid profile along with controlled body weight gain compared to untreated HFD-fed STZ group. Dayanand and Kishanchandra (2014) has also reported that the *F. carica* treatment significantly lowered the TC and TG levels in high-fat diet and Triton X-100-induced hyperlipidemic Wistar rats. Another study also confirmed the antihyperlipidemic effect of *F. carica* leaf extract on high-fat diet-induced Sprague–Dawley rats rats (Joerin et al. 2014).

The activities of key glycolytic enzymes, hexokinase, glucose-6-phosphatase and fructose-1,6-bisphosphatase were significantly altered during diabetic illness (Vestergard 1999). There is extensive evidence to show that elevated gluconeogenic enzymes such as glucose-6-phosphatase and fructose-1,6-bisphosphatase contribute to hyperglycemia in the diabetic state (Saxena et al. 1984). *F. carica* significantly normalized the activities of these gluconeogenic enzymes in the HFD-fed STZ-treated diabetic rats. *F. carica* has protective effect against the disturbed carbohydrate metabolic enzymes; this is probably due to enhanced insulin action. The insulin sensitization activity of *F. carica* was further substantiated via its improved hepatic glycogen content in the treated diabetic rats. Insulin plays a role in regulating glycogen metabolism through activation or inhibition of several mediatory enzymes and proteins (Ferrer et al. 2003).

The immunohistochemical study clearly indicated that the HFD-fed STZ-induced diabetic control rats had decreased insulin-immunoreactive expression. A defect in insulin action along the peripheral tissues resulting in hyperinsulinaemia eventually damages the structural integrity and functional status of β-cells (Yuan et al. 2001). The increase in insulin-immunoreactive expression in *F. carica-*treated diabetic rats proved the cytoprotective role of *F. carica* in preventing the deterioration of β-cells.

## Conclusions

In conclusion, the ethyl acetate extract of *F. carica* leaves possesses antidiabetic activity by stimulating the insulin production from the regenerated beta cells of the pancreas. Increased insulin secretion after treatment with *F. carica* positively altered the deranged carbohydrate metabolism in the diabetic rats by decreasing gluconeogenesis and increasing glycolysis, ultimately decreasing hyperglycemia.
